# Effect of hypergravity on the biomechanics of the musculoskeletal system in human lumbar spine: a numerical study

**DOI:** 10.3389/fbioe.2026.1842626

**Published:** 2026-06-16

**Authors:** Bing Qin, Lu Zhou, Xiaoyu Zhang, Yuheng Liu, Zhiyu Qian, Qiaoqiao Zhu

**Affiliations:** 1 Department of Biomedical Engineering, Nanjing University of Aeronautics and Astronautics, Nanjing, China; 2 College of Information Engineering, Taizhou University, Taizhou, China

**Keywords:** biomechanics, finite element analysis, hypergravity, lumbar spine, musculoskeletal modeling

## Abstract

The aim of this study was to investigate the effects of hypergravity on the biomechanics of the human lumbar spinal musculoskeletal system. We quantitatively analyzed the biomechanics of the intervertebral disc (IVD), lumbar spinal muscles, and ligament biomechanics changes in jet pilots under various hypergravity conditions, namely, 1G (represents the normal gravity on the Earth), 3G, 6G and 9G (represents various hypergravity), strengthened 10%-3G/6G/9G (various hypergravity after muscle strengthened by 10%), strengthened 20%-3G/6G/9G (various hypergravity after muscle strengthened by 20%) using a full body musculoskeletal modeling approach, and whether these biomechanical changes lead to lumbar IVD damages with a finite element method. The compressive and shear forces on the lumbar IVD, the height and water content of the lumbar IVD, muscle activation, and ligament force, as well as the mechanical damage in the IVD under the above various hypergravity were quantitatively compared and analyzed. Compared with 1G, hypergravity produced dose-dependent changes in lumbar spine biomechanics and IVD integrity. Under 3G, 6G, and 9G, average lumbar IVD compressive forces increased by 68%, 190%, and 311%, while shear forces increased by 53%, 140%, and 212%, respectively. IVD height decreased by 3.0%, 8.0%, and 12.4%, and water content decreased by 2.4%, 7.2%, and 12.4%. Most lumbar muscle activations increased by 3.0%, 7.0%, and 13.0%, whereas most ligament forces decreased by 25.0%, 34.0%, and 40.0%. AF damaged volume fraction increased by 0.5%, 3.7%, and 7.3%. IVD stress also increased markedly, with AF stress rising by 73%, 215%, and 358%, and NP stress by 58%, 163%, and 267% under 3G, 6G, and 9G, respectively. The muscle strengthening after training showed no significant change on the loading distribution among different musculoskeletal segments under hypergravity. Our results indicated that loading on the IVD significantly increased, biochemical environment and morphology within the IVD changed, and the risk of annulus fibrosus damage increased; muscle strengthening did not have a significant impact on the load on various tissues under hypergravity. Our findings were important for understanding the hypergravity-induced effect on the health of human lumbar spine.

## Introduction

With the development of high-performance jets, the resulting hypergravity environment (high G-load) has caused significant adverse effects on the health of jet pilots. It was reported that the incidence of low back pain in jet pilots was 64%, which was significantly higher than that of transport helicopter pilots (22.3%) (Grossman et al., 2012). Low back pain and lumbar intervertebral disc (IVD) damage have been the main reasons pilots being disqualified from flying (Mccrary and Syoc, 2002). Studies have reported that jet pilots experienced acute low back pain during training under an average G-load of 6.7 G (Wagstaff et al., 2012). Another study reported that 89% of jet pilots experienced muscle pain during mission (Kikukawa et al., 1995). Early signs of degeneration were more common in the spine of jet pilots than those in the general population (Hendriksen and Holewijn, 1999). It is well known that lumbar IVD damage and/or degeneration is a major contributor to low back pain (Clark and Horton, 2018; Hartvigsen et al., 2018; Urban and Roberts, 2003). Furthermore, numerous studies have indicated that abnormal mechanical loading increased the risk of lumbar IVD damage and/or degeneration (Stokes and Iatridis, 2004; Setton and Chen, 2006; Vergroesen et al., 2015). The hypergravity during jets mission ranges from 3G to 9G (Pollock et al., 2021). The reasons for the high incidence of low back pain experienced under hypergravity during jet missions were not yet fully understood.

One important factor is the sudden change in the mechanical environment of lumbar spine under hypergravity (Swain et al., 2020). Excessive compressive force on the IVD increased the risk of IVD herniation, which was a major clinical cause of low back pain (Mcgill, 2001), and spinal loading would increase in individuals with low back pain during load bearing tasks (Marras et al., 2001). Although many studies have focused on low back pain in jet pilots under hypergravity, how hypergravity quantitatively affected the mechanical environment of the lumbar spine remained largely unknown.

In addition, the muscles and ligaments within the lumbar spine were crucial for spinal stability and integrity. Studies suggested that lumbar spinal muscle strengthening training could effectively alleviate low back pain symptoms in jet pilots (Yang et al., 2021; Mendes et al., 2024). However, these studies focused primarily on low back pain symptoms and the symptomatic effects of muscle strengthening training in pilots, without quantitatively assessing how hypergravity affects IVD loading, water content, muscle activation, ligament forces, or IVD damage. The specific question we tried to solve was how hypergravity affects the biomechanics of the lumbar spinal segments (including loading on the lumbar IVD, muscle activations, and ligament forces) and the IVD damages.

**FIGURE 1:**
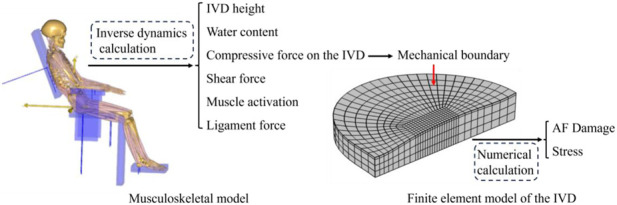
Flowchart of the coupled musculoskeletal modelling and finite element analysis.

Thus, in this study, we aimed to quantify the biomechanical responses of the human lumbar spinal musculoskeletal system under various hypergravity conditions with numerical methods. To the best of our knowledge, it is the first study on combining the musculoskeletal modelling and finite element analysis to quantify biomechanical signals change (including the loading on the IVD, water content and height of the IVD, muscle activation, ligament force, stress within the IVD) and IVD damage under hypergravity (Figure 1). This study is important for elucidating the impact of hypergravity on the biomechanical health of the lumbar spine.

## Methods

### Lumbar spine musculoskeletal biomechanics model

The effects of hypergravity on the biomechanical loadings on the lumbar spine, including the loading on the lumbar IVDs, IVD swelling (water content and height), muscle activation, and ligament forces in the lumbar spine, were studied using a full body musculoskeletal model developed with the AnyBody Modeling System (AnyBody Technology, Version 7.3, Denmark).

The anatomical structure and sizes of the body segments were scaled to a male with a height of 1.72 m and a weight of 72 kg, which was based on the average size of Chinese jet pilots (Wang et al., 2022). Specifically, five lumbar IVDs (L1L2, L2L3, L3L4, L4L5, and L5S1), five lumbar vertebrae (vertebra L1-L5), six lumbar ligament groups [including anterior longitudinal ligament (ALL), posterior longitudinal ligament (PLL), interspinous (IS),supraspinous (SS), flavum (FL), and intertransverse (IT)] and ten major muscle groups [including lumbar multifidus (MF), erector spinae (ES), psoas major (PM), quadratus lumborum (QL), obliquus externus (OE), obliquus internus (OI), semispinalis (SR), thoracic multifidus (TMF), rectus abdominis (RA), and transversus abdominis (TRA)] were included in the human lumbar spine in this model as described in our previous publication (Wu et al., 2023).

Following established methods, the lumbar vertebrae were modeled as rigid bodies, and the lumbar IVD were modeled as 6 degrees of freedom joints with linear momentum-rotational deformation and linear force-translational deformation relationships (Baldoni and Gu, 2019):

$$M_{i}=h_{i}\cdot \theta_{i}$$ (1)

$$F_{i}=k_{i}\cdot x_{i}$$ (2)

where M_i_ represents the reaction moment on the lumbar IVD, θ_i_ is the rotational angle along the i^th^ axis, h_i_ is the rotational stiffness of the lumbar IVD, F_i_ represents the reaction force on the lumbar IVD, x_i_ is the translational displacement along the i^th^ axis, k_i_ is the translational stiffness of the lumbar IVD, with values from the literature (Baldoni and Gu, 2019).

To simulate the changes of the morphology (the height of the IVD) and water content within the lumbar IVD in hypergravity, we used a deformation-dependent swelling pressure model based on the framework developed by Lai et al. (Lai et al., 1991), as implemented in our previous work (Wu et al., 2023; Baldoni and Gu, 2019; Qin et al., 2022). This model accounts for the interplay between swelling pressure, fixed charge density (FCD), and IVD deformation to predict changes in height and water content of the lumbar IVD under mechanical loading. The deformation dependent swelling pressure of the IVD was introduced by the following equation (Lai et al., 1991):

$$F_{s}=RT\left(\sqrt{{c^{F}}^{2}+{4c^{*}}^{2}}-2c^{*}\right)$$ (3)

where R is the universal gas constant (8.3144 JK^-1^mol), T is the temperature in Kelvin (310.15 K), $c^{*}$ is the concentration of Na^+^ and Cl^−^ in the surrounding environment of the discs (150 mM). $c^{F}$ is the FCD inside the IVD, which is dependent on IVD deformation as follow (Lai et al., 1991):

$$c^{F}=c_{0}^{F}\frac{\phi_{0}^{w}}{\phi_{0}^{w}+J-1}$$ (4)

where $c_{0}^{F}$ is FCD inside the IVD at reference state, J is the volume ratio of IVD between deformed and reference state. Assuming that during swelling, the percentage changes in IVD dimension were similar in all three principal directions, the volume ratio was estimated by:

$$J={\left(h/h_{0}\right)}^{3}$$ (5)

where h is IVD height after deformation and h_0_ is IVD height at reference state. The water content in the IVD is deformation dependent and is calculated as follow (Lai et al., 1991):

$$\phi^{w}=\frac{\phi_{0}^{w}+J-1}{J}$$ (6)

where $\phi^{w}$ is IVD water content after deformation, and $\phi_{0}^{w}$ is IVD water content at the reference state. The compressive loading on the IVD (F) due to body weight, muscle forces, and ligament forces (in direction perpendicular to the lower surface of the IVD) was assumed to consist of two forces, namely, a swelling force ( $F_{S}$ ) generated by the swelling pressure, and an elastic force ( $F_{E}$ ) generated by IVD deformation. It was calculated as:

$$F=F_{S}+F_{E}$$ (7)

In this study, the average FCD in annulus fibrosus (AF) was assumed to be 80% of that in the nucleus pulposus (NP) for healthy IVD based on experimental data (Urban and Maroudas, 1979), and the cross-sectional area of NP was assumed to be 40% of the whole IVD cross-sectional area, also based on experimental data (Pooni et al., 1986). The swelling pressure in the lumbar IVD was estimated as:

$$F_{S}=A_{IVD}RT\left[0.4\left(\sqrt{{c_{NP}^{F}}^{2}+{4c^{*}}^{2}}-2c^{*}\right)+0.6\left(\sqrt{{c_{AF}^{F}}^{2}+{4c^{*}}^{2}}-2c^{*}\right)\right]$$ (8)

where $A_{IVD}$ is the IVD cross-sectional area, $c_{NP}^{F}$ is the mean FCD in the NP, and $c_{AF}^{F}$ is the mean FCD in the AF. The average water content in the IVD was estimated by: $\phi^{w}=0.4\phi_{NP}^{w}+0.6\phi_{AF}^{w}$ , where $\phi_{NP}^{w}$ and $\phi_{AF}^{w}$ are the water content in the NP and AF, respectively. The water content within the lumbar IVD were set to be 0.85 for NP and 0.775 for AF (Wu et al., 2023), the IVD height were set to be 9 mm, 10.4 mm, 11.5 mm, 11.8 mm, and 11.3 mm for L1L2 IVD, L2L3 IVD, L3L4 IVD, L4L5 IVD, and L5S1 IVD (Wu et al., 2023) at the reference state.

Ligament forces were computed using force-strain relationships derived from experimental data. The spinal ligaments were considered to have piecewise linear mechanical properties, that the stiffness of each ligament was dependent on its strain (Baldoni and Gu, 2019; Chazal et al., 1985). The strain was calculated from the deformation of its insertion points. For the modeling of the lumbar spinal muscles, maximum muscle strength was proportional to its functional cross-sectional area (FCSA) (Zee et al., 2007).

**FIGURE 2:**
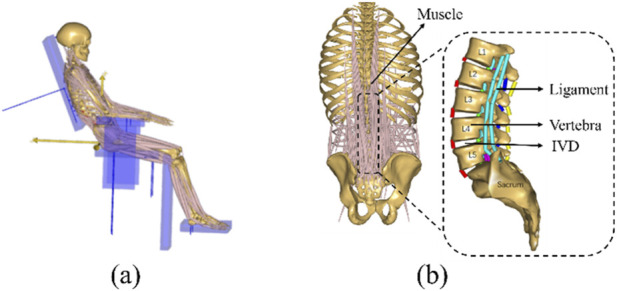
**(a)** Posture in gravity and various hypergravity established in this study and **(b)** Schematic of the human lumbar spine showing vertebrae, lumbar intervertebral disc, major muscles and ligaments.

In this study, a typical jet pilot sitting posture was developed using AnyBody Modeling System to simulate the pilot’s lumbar spine biomechanics during flight tasks (Kong, 2017). The full-body musculoskeletal geometry (Figure 2) was from the Anybody database (AMMR2.3.4) which were originally developed by Zee et al. (2007). A rigid connection was established between the pilot and the seat (Kong, 2017).

To explore the effects of various levels of hypergravity on the musculoskeletal system of the human lumbar spine, the biomechanics of the lumbar spine under 1G, 3G, 6G, 9G as well as 3G, 6G and 9G following muscle training were analyzed. In this musculoskeletal model, muscle strengthening was modeled by increasing the FCSA of the muscles by 10% and 20% (In this study, the FCSA is linearly correlated with the maximal strength of muscles) (Shigaki et al., 2018). We quantitatively compared and analyzed the compressive and shear forces on the lumbar IVD, water content and height of the lumbar IVD, muscle activations and ligament forces between 1G and various hypergravity conditions.

### Intervertebral disc mechanical damage model

The effects of hypergravity on the mechanical damage of the IVD were studied using a finite element model developed based on the multiphasic mixture theory (Gu et al., 1998; Sun et al., 1999). The lumbar IVD was modeled as a multiphasic mixture consisting of a porous solid phase, an interstitial fluid phase, and a solute phase with multiple species, including charged (e.g., sodium ion, chloride ion) and uncharged (e.g., glucose, oxygen, and lactate) solutes (Mow et al., 1980). The AF consists of a homogenous matrix with two kinds of fiber bundles embedded in it. The homogenous matrix was modeled as hyperelastic materials, its strain energy density function is as follows (Gao et al., 2017):

$$w_{AF}=\left(1-d^{m}\right)w_{m}+\sum_{\vartheta =1,2}\left(1-d^{\theta }\right)w_{f}^{\theta }$$ (9)

where $d^{m}$ is the damage parameter of the AF matrix. Since the contribution of fiber bundles to the tensile modulus of AF is much larger than that of the ground matrix, and the yield strain of fiber bundles under tension is much smaller than that of the ground matrix (Ebara et al., 1996; Fujita et al., 1997), the damage of matrix is ignored ( $d^{m}$ = 0). $w_{m}$ is the strain energy density of the matrix of AF, $d^{\theta }$ is the damage variable of the two fiber bundles in the AF, and $w_{f}^{\theta }$ is the strain energy density of the two fiber bundles. The damage variable for fiber bundles is as follows (Gao et al., 2017; Balzani et al., 2006; Famaey et al., 2013):

$$d^{\theta }=d_{\max}\left[1-\exp\left(-\frac{\beta^{\theta }}{\gamma }\right)\right]$$ (10)

where $d_{\max}$ (∈ [0,1]) and γ are two damage parameters, and $\beta^{\theta }$ is the internal damage variable which is a function of $w_{f}^{\theta }$ . The parameter $\beta^{\theta }$ is defined as (Balzani et al., 2006; Famaey et al., 2013):

$$\beta^{\theta }=\max_{0\leq t\leq \tau }\langle \;w_{f}^{\theta }\left(\tau \right)-{\hat{w}}_{f}\rangle$$ (11)

where ${\hat{w}}_{f}$ is the threshold value of the strain energy density above which damage would occur. The bracket, $\langle ·\rangle$ , defined as $\langle ·\rangle$ = 0.5 [( · ) + $\left|·\right|$ ], is used to filter out negative values. In this study, an overall damage variable is defined to evaluate the damage of both families of fiber bundles by,

$$d_{AF}=\frac{1}{2}\sum_{\theta =1,2}d^{\theta }$$ (12)

${\hat{w}}_{f}$ is set to be 100 kJ/m^3^ (Ebara et al., 1996), $d_{\max}$ and γ were set to be 0.5 and 50 kJ/m^3^ (Pezowicz, 2010).

We developed a 3D finite element model based on the theoretical framework with COMSOL software (COMSOL, Inc., United States). The geometry of the lumbar IVD was generated based on a L4-L5 human disc (Gao et al., 2017). The values of the Lame constants (λ and μ) in NP were λ = 0.04 MPa and μ = 0.06 MPa; in AF, λ was linearly increased from 0.04 to 0.17 MPa and μ linearly increased from 0.06 to 0.14 MPa from the innermost AF region to the outermost AF regions based on experimental results, respectively (Gao et al., 2016).

The mechanical boundary condition for the lumbar IVD damage model was obtained from results of the lumbar spine musculoskeletal model calculated above. The biomechanical response within the lumbar IVD under various hypergravity conditions, including 1G (represents the normal Earth gravity), 3G (represents hypergravity conditions, which is corresponding to three times the Earth’s normal gravity), 6G (represents hypergravity conditions, which is corresponding to six times the Earth’s normal gravity), strengthened-3G (under 3G hypergravity after muscle strengthened 10%), and strengthened-6G (under 6G hypergravity after muscle strengthened 10%), were analyzed. All outputs (compressive force, shear force, IVD height, water content, muscle activation, ligament force, AF damage, stress) under each hypergravity and each strengthening condition (10% and 20%) were compared with those under 1G. All outputs were expressed as absolute values.

### Model validation

The musculoskeletal model has been validated against *in vivo* pressure measurements under different daily postures (e.g., lying supine, sitting slouched, sitting straight, standing, standing with flexion, standing with weight lifting) as reported in our previous publication (Qin et al., 2022; Hans-Joachim et al., 2001). The simulated compressive forces on the lumbar IVD at 1G showed good agreement with the experimental data, with an accuracy of 90% across the 12 daily activities.

This IVD damage model based multiphasic mixture theory has been previously validated against experimental data from literature and can well characterize the occurrence and development of the damage in the AF under various compressive loading (Gao et al., 2017). Briefly, this IVD damage model has been previously validated for simulating the stretch and damage of fiber bundles within the lumbar IVD under compressive loadings against experimental data from literature (Ebara et al., 1996; Skaggs et al., 1994).

## Results

### Compressive forces on the IVD

**FIGURE 3:**
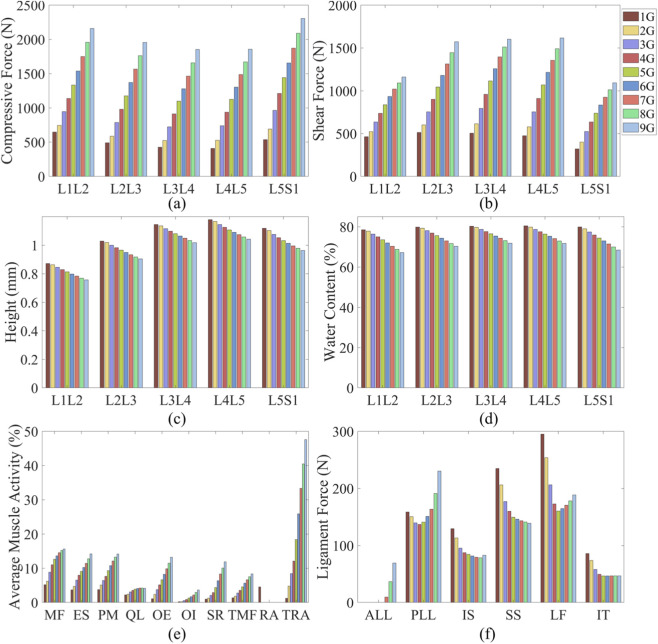
Biomechanical changes in the lumbar spine under normal gravity (1G) and various hypergravity (2G,3G,4G,5G, 6G,7G,8G, 9G). **(a)** Compressive forces on lumbar intervertebral disc (IVD) from L1L2 to L5S1. **(b)** Shear forces on the lumbar IVD. **(c)** IVD height. **(d)** Water content within the Iumbar IVD. **(e)** Average muscle activation for ten muscle groups: Multifidus (MF), erector spinae (ES), psoas major (PM), quadratus lumborum (QL), obliquus externus (OE), obliquus internus (OI), semispinalis (SR), thoracic multifidus (TMR), rectus abdominis (RA) and transversus abdominis (TRA). **(f)** Ligament forces for six ligaments: anterior longitudinal ligament (ALL), posterior longitudinal ligament (PLL), interspinous (IS), supraspinous (SS), flavum (LF), and intertransverse (IT).

**FIGURE 4:**
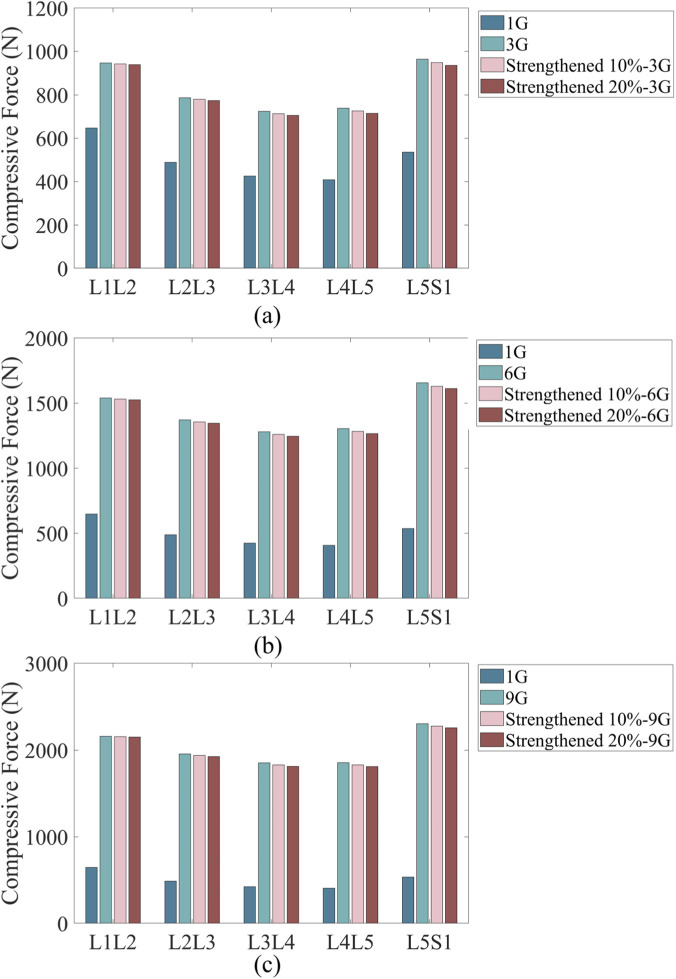
Comparison of compressive forces on lumbar intervertebral disc under gravity (1G) and various hypergravity. **(a)** 1G,3G, strengthened 10%-3G and strengthened 20%-3G. **(b)** 1G, 6G, strengthened 10%-6G, strengthened 20%-6G, **(c)** 1G, 9G, strengthened 10%-9G, strengthened 20%-9G.

Compared to 1G, the average compressive force (in Newtons, N) on the lumbar IVD across all five levels (L1L2 to L5S1) increased by 68% under 3G, increased by 190% under 6G, and increased by 311% under 9G (Figure 3a). The increase in compressive force induced by hypergravity was reduced by approximately 2% and 5% under strengthened 10% and 20%, respectively (Figure 4).

### Shear forces on the IVD

**FIGURE 5:**
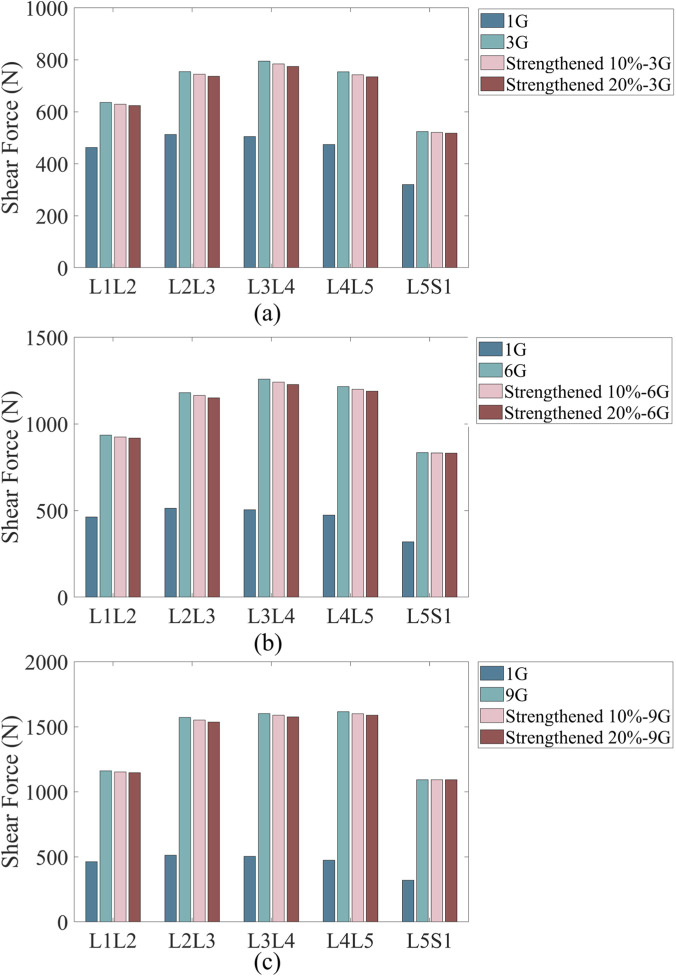
Comparison of shear forces on lumbar intervertebral disc under gravity (1G) and various hypergravity. **(a)** 1G,3G, strengthened 10%-3G and strengthened 20%-3G. **(b)** 1G, 6G, strengthened 10%-6G, strengthened 20%-6G, **(c)** 1G, 9G, strengthened 10%-9G, strengthened 20%-9G.

Compared to 1G, the average shear force (N) on the lumbar IVD increased by 53% under 3G, increased by 140% under 6G, and increased by 212% under 9G (Figure 3b). The increase in shear force induced by hypergravity was reduced by 2% and 4% under strengthened 10% and 20%, respectively (Figure 5).

### IVD height

**FIGURE 6:**
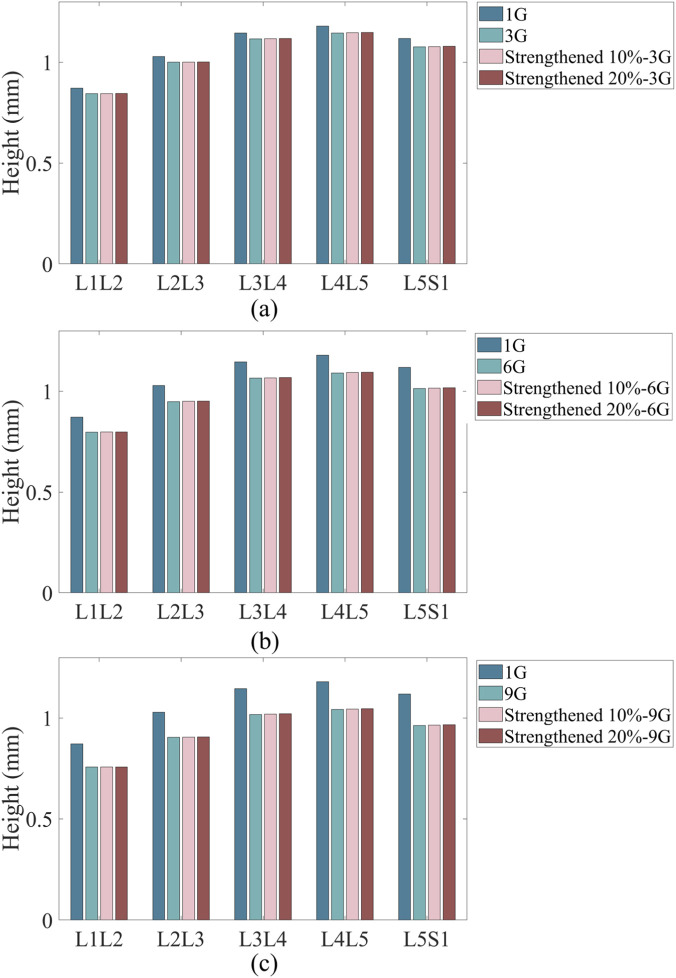
Comparison of lumbar intervertebral disc height under gravity (1G) and various hypergravity. **(a)** 1G,3G, strengthened 10%-3G and strengthened 20%-3G. **(b)** 1G, 6G, strengthened 10%-6G, strengthened 20%-6G, **(c)** 1G, 9G, strengthened 10%-9G, strengthened 20%-9G.

Compared to 1G, the average height (mm) of the lumbar IVD decreased by 3.0% under 3G, decreased by 8.0% under 6G, and decreased by 12.4% under 9G (Figure 3c). The decrease in IVD height induced by hypergravity was reduced by 3% and 5% under strengthened 10% and 20%, respectively (Figure 6).

### Water content

**FIGURE 7:**
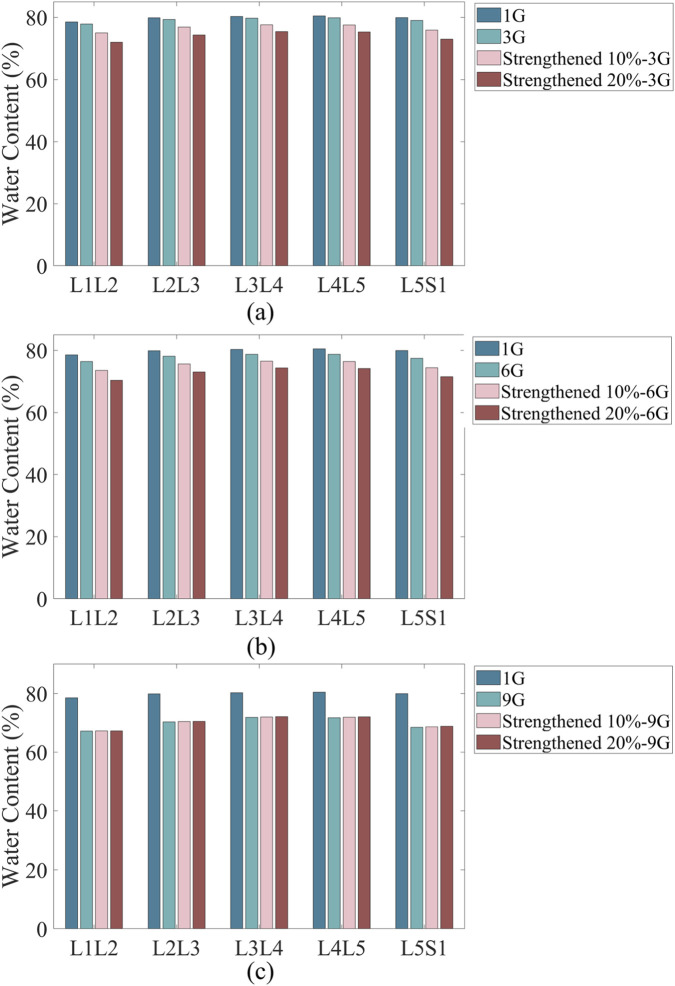
Comparison of water content of lumbar intervertebral disc under gravity (1G) and various hypergravity. **(a)** 1G,3G, strengthened 10%-3G and strengthened 20%-3G. **(b)** 1G, 6G, strengthened 10%-6G, strengthened 20%-6G, **(c)** 1G, 9G, strengthened 10%-9G, strengthened 20%-9G.

Compared to 1G, the average water content (dimensionless, 0%–100%) of the lumbar IVD decreased by 2.4% under 3G, decreased by 7.2% under 6G, and decreased by 12.4% under 9G (Figure 3d). The decrease in water content induced by hypergravity was reduced by 3% and 5% under strengthened 10% and 20%, respectively (Figure 7).

### Muscle activation

**FIGURE 8:**
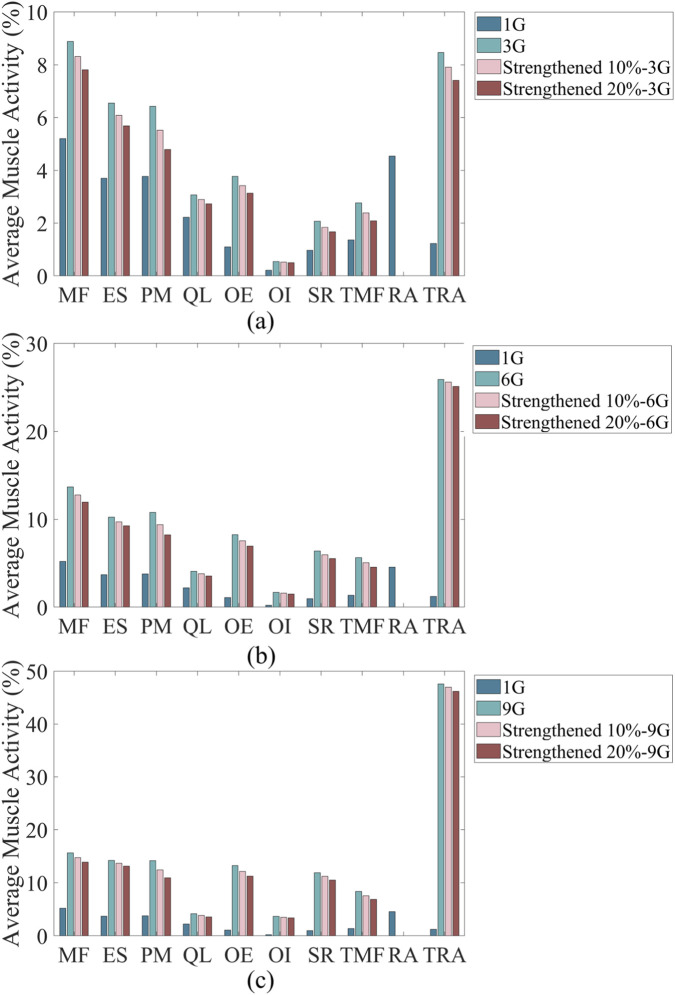
Comparison of average muscle activation under gravity (1G)and various hypergravity. **(a)** 1G,3G, strengthened 10%-3G and strengthened 20%-3G. **(b)** 1G, 6G, strengthened 10%-6G, strengthened 20%-6G, **(c)** 1G, 9G, strengthened 10%-9G, strengthened 20%-9G.

Compared to 1G, the average activation (dimensionless, 0%–100%) of most lumbar spinal muscles increased by 3% under 3G, increased by 7% under 6G, and increased by 13% under 9G (Figure 3e). The increase in muscles activation induced by hypergravity was reduced by 1% and 2% under strengthened 10% and 20%, respectively (Figure 8).

### Ligament force

**FIGURE 9:**
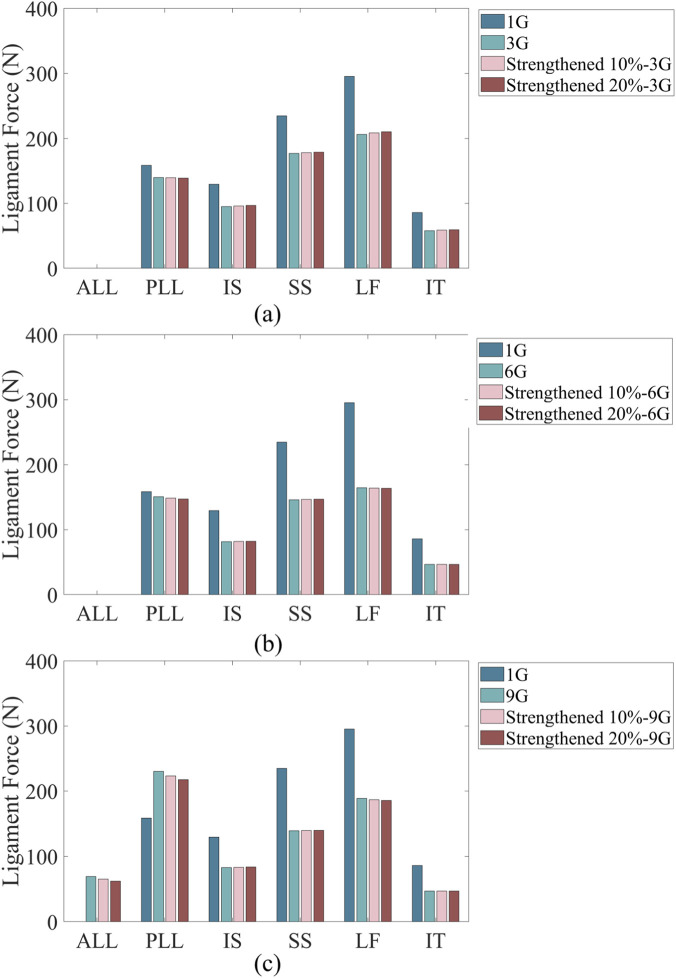
Comparison of ligament forces in lumbar spine under gravity (1G) and various hypergravity. **(a)** 1G,3G, strengthened 10%-3G and strengthened 20%-3G. **(b)** 1G, 6G, strengthened 10%-6G, strengthened 20%-6G, **(c)** 1G, 9G, strengthened 10%-9G, strengthened 20%-9G.

Compared to 1G, the average force (N) in most ligaments decreased by 25% under 3G, decreased by 34% under 6G, and decreased by 40% under 9G (Figure 3f). The decrease in ligament force induced by hypergravity was reduced by 1% and 2% under strengthened 10% and 20%, respectively (Figure 9).

### Biomechanic within the lumbar IVDs

**FIGURE 10:**
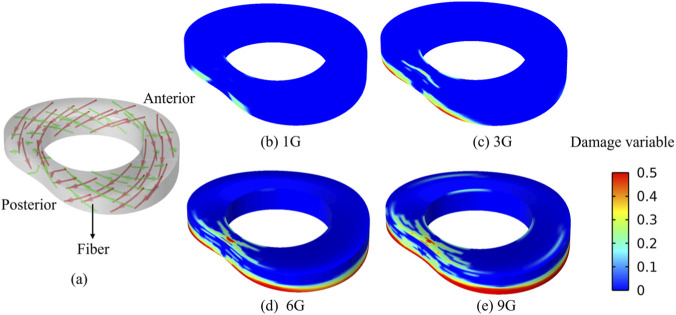
**(a)** Schematic of the annulus fibrosus and 3D distributions of damage (represented by the damage variable defined in Equation. 12, which ranges from 0 to 1; 0 indicates no damage, while higher values indicate more severe damage) within the annulus fibrosus under different gravity conditions, **(b)** 1G, **(c)** 3G, **(d)** 6G, and **(e)** 9G.

Damage within the AF: Compared to 1G, the volume fraction of the damage (dimensionless, 0%–100%) in the AF (relative to the whole AF) increased under hypergravity conditions. The volume fraction of the damage in the AF was 0 under 1G, it increased by 0.5%, 3.7%, and 7.3% under 3G, 6G, and 9G compared to that under 1G, respectively (Figure 10).

**FIGURE 11:**
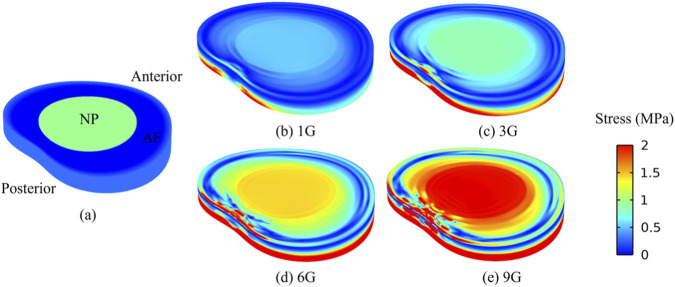
**(a)** Schematic of the intervertebral disc and 3D distributions of stress (Von Mises stress) within the intervertebral disc (IVD) under **(b)** 1G, **(c)** 3G, **(d)** 6G, and **(e)** 9G. (NP, nucleus pulposus; AF, annulus fibrosus).

Stress within the lumbar IVD: Compared to 1G, the stress (MPa) within the lumbar IVD increased under hypergravity conditions. It increased by 73%, 215%, and 358% in AF, and it increased by 58%, 163%, and 267% in NP under 3G, 6G, and 9G compared to that under 1G, respectively. The maximum stress was distributed in the posterior of AF under 1G, while it was distributed in the NP and the posterior of AF under 9G (Figure 11).

## Discussion

In this study, we quantitatively analyzed the biomechanics of the lumbar spine, including the biomechanical changes in the lumbar IVDs, muscles, and ligaments in jet pilots, under various hypergravity conditions (3G, 6G, 9G, with and without 10% and 20% muscle strengthening) using a full body musculoskeletal model, and analyzed whether these biomechanical changes are associated with lumbar IVD damages with a finite element method. We found that the compressive and shear forces on the lumbar IVD and activation of most muscles increased under hypergravity conditions compared to those under 1G. Furthermore, these increases were more remarkable at higher degrees of hypergravity. In contrast, the height and water content of most lumbar IVDs and forces in most ligaments decreased, and the changes became more remarkable at higher degrees of hypergravity. We also found that muscle strengthening (10% and 20%) showed no significant change on the loading distribution among different musculoskeletal segments under hypergravity compared to non-strengthened conditions. Our results showed that the mechanical damage within the AF and the stress within the lumbar IVD were larger under hypergravity conditions than those under 1G. The damage was observed to initiate in the outer posterior region of the AF, and the posterolateral AF was identified as the most affected region, consistent with clinical observations. To the best of our knowledge, this is the first numerical study that quantitatively analyzed the effect of hypergravity on the biomechanics of the human lumbar spine in jet pilots.

The compressive and shear forces on the lumbar IVD increased under hypergravity (e.g., 2G, 3G, …, 9G) compared to those under 1G. For example, the compressive force on the lumbar IVD under 6G was equivalent to the compressive forces on the lumbar IVD when standing flexed forward at 38° on the earth (under 1G), the compressive force on the lumbar IVD under 9G was equivalent to the compressive forces on the lumbar IVD when lifting a 20 kg weight while standing flexed forward at 38° on the earth (Qin et al., 2022). This is because the compressive and shear forces on the lumbar IVD were a combination of body weight, muscle forces and ligament forces. In hypergravity, the gravitational loading on all tissues of human were increased, leading to an increase in the mechanical loading on the lumbar IVD, which results in an increase in compressive and shear forces on the lumbar IVD. Our results showed that the damage within the AF and the stress within the lumbar IVD increased under hypergravity compared to 1G, which suggested that excessive loading caused by hypergravity may affect the mechanical environment within the lumbar IVD and may increase the risk of the lumbar IVD damage (Stokes and Iatridis, 2004; Gallagher and Marras, 2012). The damage was seen initiated from the outer posterior area of the AF, and the damage region increased significantly under hypergravity compared to 1G. Therefore, the posterolateral AF might be the region most susceptible to damage under hypergravity. Studies reported that jet pilots have higher incidence of lumbar IVD damages (Zhang et al., 2021). Numerous studies showed that lumbar IVD damage was a major cause of low back pain (Wang et al., 2024), and low back pain has been affecting the health and ability to perform tasks for jet pilots. These findings are helpful for better understanding why jet pilots have higher incidence of lower back pain.

The water content and height of the lumbar IVDs significantly decreased under hypergravity conditions compared to those under 1G. On Earth, the swelling pressure and elastic forces within the lumbar IVDs balanced the mechanical loading exerted on the lumbar IVD by the muscle, ligament, body weight, etc. Hypergravity induced much larger compressive loading on the lumbar IVD, leading to water and height loss, which was consistent with results in the literature that excessive compressive loading would lead to water loss within the lumbar IVD (Mcmillan et al., 1996). The water within the lumbar IVD played a crucial role in maintaining the normal biochemical environment and biomechanical function of the lumbar IVD. Based on previous studies (Gu and Yao, 2003; Yao and Gu, 2007), a decrease in water content may reduce the transport rate of solutes within the IVD, including the rate of nutrient supply and metabolic waste efflux, and may affect the metabolic balance within the lumbar IVD (Gu et al., 2014). Moreover, the water within the lumbar IVD was essential for supporting the mechanical loading and providing the flexibility for the spine. When water content decreased, fluid pressure decreased, and a greater share of the loading shifts to the solid matrix, increasing local stresses. These elevated mechanical stresses may accelerate matrix damage and increase the risk of lumbar IVD degeneration, which was consistent with the reports in the literature (Takashima et al., 2012; Zhu et al., 2014).

The activation of most muscles increased under hypergravity conditions compared to 1G, and these increases were more remarkable at higher degrees of hypergravity. This was because the loading on the lumbar spinal muscles increased under hypergravity, and the muscles were activated by high intensity to maintain the stability of the lumbar spine. Studies using parabolic flight to simulate the effects of hypergravity on human also showed a significant increase in the activation of the TRA (increased by 47% under hypergravity), ES (increased by 41% under hypergravity) and MF (increased by 57% under hypergravity) under hypergravity (Swanenburg et al., 2020).

Forces in most ligaments decreased under hypergravity conditions compared to those under 1G, these decreases were more remarkable at higher degrees of hypergravity. This is because the height of the lumbar IVD decreased under hypergravity, and led to a laxity of the ligaments, resulting in the decrease in ligament forces. Ligaments, muscles, and lumbar IVDs collectively play a crucial role in bearing and transmitting mechanical loads and maintaining spinal stability. Studies have found that the decrease in IT ligament force led to a significant increase in the mechanical loading on the lumbar IVD and stress within the lumbar IVD, thus increasing the risk of the lumbar IVD damage and reducing the stability of the lumbar spine (Zhang et al., 2022). This is consistent with our results that the mechanical loading on the ligament decreased while the loading on the lumbar IVD and the stress within the lumbar IVD increased under hypergravity. However, the forces in the ALL and PLL increase slightly at some hypergravity conditions, possibly due to extreme spinal shear deformation. Overall, most ligaments were not stretched and their forces decreased under hypergravity. These results reflected the physiological role of ligaments in limiting excessive motion.

Muscle strengthening had no significant effect on the changes in the mechanical loading among lumbar spine musculoskeletal system under hypergravity. Pilots could strengthen their muscle through core stabilization exercises, resistance training (e.g., deadlifts, back extensions), and functional movements targeting the lumbar extensor and abdominal muscles (Mendes et al., 2024; Shigaki et al., 2018). In this model, strengthening was simulated by increasing the functional cross-sectional area (FCSA) by 10% and 20%, which proportionally increased the maximum force of muscle. Our results suggested that strengthened muscle after training could lead to a decrease in muscle activation under hypergravity, which was consistent with the results reported in the literature that muscle strengthening training could reduce the muscle activation under hypergravity (Rausch et al., 2021). The loads on other tissues (e.g., IVD) did not change significantly, which suggests that the load on the muscles also remained essentially unchanged. This is because the net joint moment required to maintain the fixed seated posture under hypergravity did not change; a stronger muscle can generate the same muscle force with lower activation. The increase in loading on the other musculoskeletal system of the lumbar spine such as the compressive and shear forces on the lumbar IVD caused by hypergravity was difficult to effectively mitigate or offset through muscle strengthening alone. Our study showed that muscle strengthening did not significantly reduce the loading on the IVD or ligament under hypergravity. It clarified the biomechanical limits of muscle strengthening as a standalone countermeasure. Additionally, our results showed that muscle activation decreased after strengthening (Figure 8), which might reduce muscle fatigue–a known contributor to low back pain. This provides a plausible mechanism for symptom relief without reducing the loading on the IVD. Therefore, muscle strengthening could be considered as part of a broader countermeasure strategy (Rausch et al., 2021).

This study has some limitations. First, currently the model only considered the transient effects of the impact loading induced by hypergravity on the lumbar musculoskeletal system, the long-term effects of hypergravity on the lumbar musculoskeletal system were not included in our model yet. The observed changes in loading on the lumbar spine may not fully represent the state of the lumbar spine under prolonged exposure. Specifically, the model may overestimate the loading on the lumbar spine, as long-term adaptation could lead to a change of loading distribution. Second, in order to explore the effect of muscle strengthening on the changes in loading among lumbar spine segments under hypergravity, we assumed 10% and 20% strengthening for all lumbar spinal muscles. However, different muscles of jet pilots may experience different degrees of strengthening in actual training. This assumption may affect the simulated magnitude of the loading on each muscle groups, but it may not affect the loading on other lumbar tissues, since muscle strengthening showed no significant effect on the changes in the loading magnitudes among lumbar spine musculoskeletal system under hypergravity.

Based on the limitations and findings of this study, future research could address several directions. First, a long-term adaptive model incorporating muscle plasticity and IVD creep is needed to develop to simulate the long-term response exposure under hypergravity. Second, based on the findings that the posterolateral AF was the most damaged region, imaging studies in jet pilots could validate whether simulated damage locations correspond to actual IVD damage. Finally, muscle strengthening alone did not significantly reduce the loading on the IVD, future studies could test alternative countermeasures (e.g., anti-G suits, lumbar supports).

In conclusion, the effects of hypergravity conditions on the biomechanics of human lumbar spinal musculoskeletal system were quantitatively analyzed with numerical methods. We found that hypergravity had significant effects on the mechanical loading on the lumbar IVD (increase), the height (decrease) and water content (decrease) of most lumbar IVDs, muscle activation (increase), ligament force (decrease), the damage area of AF damage (increase), and stress (increase) within the lumbar IVD. And the muscle strengthening showed no significant effect on the mechanical loading distribution among the lumbar spinal segments under hypergravity conditions. These findings are helpful for a better understanding of how hypergravity affects the biomechanics of human lumbar spinal musculoskeletal system, which is important for understanding the hypergravity-induced effect on the health of human lumbar musculoskeletal system.

## Data Availability

The raw data supporting the conclusions of this article will be made available by the authors, without undue reservation.
