# Supplementary material for: Effect of hypergravity on the biomechanics of the musculoskeletal system in human lumbar spine: a numerical study
Source: Front Bioeng Biotechnol. 2026 Jun 16;14:1842626. doi: 10.3389/fbioe.2026.1842626 (PMC13315218; doi:10.3389/fbioe.2026.1842626)
Supplement: Supplementary file 1 [file Table1.docx]

Table 1 Parameters for Lumbar intervertebral disc height ($h_{0}$), cross-sectional area ($A_{0}$), water content ($\phi_{0}^{w}$), and fixed charge density ($c_{0}^{F}$) in the human musculoskeletal model.

|  | $h_{0}[$mm] ^1^ | $A_{0}$[mm^2^] ^2^ | $\phi_{0}^{w}$ ^3^ | | $c_{0NP}^{F}[$mol/m^3^] ^4^ |
| --- | --- | --- | --- | --- | --- |
|  |  |  | NP | AF |  |
| L1-L2 | 9 | 1425 | 0.85 | 0.775 | 261 |
| L2-L3 | 10.4 | 1658 |  |  | 242 |
| L3-L4 | 11.5 | 1714 |  |  | 239 |
| L4-L5 | 11.8 | 1684 |  |  | 215 |
| L5-S1 | 11.3 | 1709 |  |  | 217 |

NP: Nucleus pulposus, AF: Annulus fibrosus.

1. Roberts N, Gratin C, Whitehouse GH. MRI analysis of lumbar intervertebral disc height in young and older populations. J Magn Reson Imaging. 1997; 7:880–6.

2. Pooni JS, Hukins DW, Harris PF, Hilton RC, Davies KE. Comparison of the structure of human intervertebral discs in the cervical, thoracic and lumbar regions of the spine. Surg Radiol Anat 1986; 8:175–82.

3. Gu W, Zhu Q, Gao X, Brown MD. Simulation of the progression of intervertebral disc degeneration due to decreased nutritional supply. Spine 2014;39: E1411–17.

4. Urban JP, McMullin JF. Swelling pressure of the lumbar intervertebral discs: inﬂuence of age, spinal level, composition, and degeneration. Spine 1988; 13:179–87.

Table 2 Ligament stiffness values in the human musculoskeletal model.

| Ligament ^1^ | Stiffness (N/mm) | Strain (%) |
| --- | --- | --- |
| anterior longitudinal ligament (ALL) | 36.2 | <0,11> |
|  | 115.9 | <11, 41> |
|  | 43 | <41, 51> |
| posterior longitudinal ligament (PLL) | 52.7 | <0,11> |
|  | 127 | <11,28> |
|  | 37.1 | <28,37> |
| Interspinous (IS),  Supraspinous (SS) | 13 | <0,14> |
|  | 38.5 | <14,36> |
|  | 10.3 | <36,48> |
| Flavum (FL) | 23.4 | <0,8> |
|  | 54.5 | <8,20> |
|  | 12.5 | <20,25> |
| Intertransverse (IT) | 12.5 | <0,9> |
|  | 61.4 | <9,15> |
|  | 25 | <15,17> |

Chazal J, Tanguy A, Bourges M, Gaurel G, Escande G, Guillot M, et al. Biomechanical properties of spinal ligaments and a histological study of the supraspinal ligament in traction.J Biomech. 1985; 18:167–76.

Table 3 Intervertebral disc stiffness in the musculoskeletal model.

| Intervertebral disc stiffness | values |
| --- | --- |
| Flexion [N m/deg] | 0.54 ^1^ |
| Extension [N m/deg] | 0.78 ^1^ |
| Lateral Bending [N m/deg] | 0.69 ^1^ |
| Axial Rotation [N m/deg] | 2.52 ^1^ |
| Compression [N/mm] | 789 ^2^ |
| Shear [N/mm] | 245 ^3^ |

1. Schmidt, T.A., An, H.S., Lim, T.H., Nowicki, B.H., Haughton, V.M., 1998. The stiffness of lumbar spinal motion segments with a high-intensity zone in the anulus fibrosus. Spine 23, 2167-2173.

2. Pollintine, P., van Tunen, M.S., Luo, J., Brown, M.D., Dolan, P., Adams, M.A., 2010. Time-dependent compressive deformation of the ageing spine: relevance to spinal stenosis. Spine 35, 386-394.

3. Bisschop, A., Mullender, M.G., Kingma, I., Jiya, T.U., van der Veen, A.J., Roos, J.C., van Dieen, J.H., van Royen, B.J., 2012. The impact of bone mineral density and disc degeneration on shear strength and stiffness of the lumbar spine following laminectomy. European Spine Journal 21, 530-536.

Table 4. Parameters used as inputs for the calculations in the finite element model.

| Parameters | | Values |
| --- | --- | --- |
| T [K] | | 310 |
| c* [mol/m3] | | 150 ^1^ |
| C | | 0.66 |
| Elasticity constants ^2,3^  [MPa] | λ_NP_ | 0.04 |
|  | λ_AF_ | 0.04 to 0.17 |
|  | μ_NP_ | 0.06 |
|  | μ_AF_ | 0.06 to 0.14 |

1. Zhu Q, Jackson AR, Gu WY. Cell viability in intervertebral disc under various nutritional and dynamic loading conditions: 3D ﬁnite element analysis. J Biomech 2012; 45:2769–77.

2. Cortes, D.H., et al., 2014. Elastic, permeability and swelling properties of human intervertebral disc tissues: A benchmark for tissue engineering. Journal of Biomechanics 47, 2088-2094.

3. Iatridis, J.C., et al., 1999. Shear mechanical properties of human lumbar annulus fibrosus. J Orthopaed Res 17, 732-737.

Table 5 Settings of the finite element model.

| Parameters | Settings |
| --- | --- |
| Solver | COMSOL PARDISO (Direct) |
| Iteration | Fully coupled |
| Relative tolerance | 0.001 |
| Number of elements | 2674 |
